# The “obesity paradox” in patients with atrial fibrillation: Insights from the Gulf SAFE registry

**DOI:** 10.3389/fcvm.2022.1032633

**Published:** 2022-11-30

**Authors:** Yan-Guang Li, Peng-Xin Xie, Alawi A. Alsheikh-Ali, Wael AlMahmeed, Kadhim Sulaiman, Nidal Asaad, Shu-Wang Liu, Mohammad Zubaid, Gregory Y. H. Lip

**Affiliations:** ^1^Department of Cardiology and Institute of Vascular Medicine, Peking University Third Hospital, Beijing, China; ^2^College of Medicine, Mohammed Bin Rashid University of Medicine and Health Sciences, Dubai, United Arab Emirates; ^3^Heart and Vascular Institute, Cleveland Clinic, Abu Dhabi, United Arab Emirates; ^4^Department of Cardiology, Royal Hospital, Muscat, Oman; ^5^Department of Cardiology, Heart Hospital, Hamad Medical Corporation, Doha, Qatar; ^6^Department of Medicine, Faculty of Medicine, Kuwait University, Kuwait City, Kuwait; ^7^Liverpool Centre for Cardiovascular Science at University of Liverpool, Liverpool Heart and Chest Hospital, Liverpool John Moores University, Liverpool, United Kingdom; ^8^Department of Clinical Medicine, Aalborg University, Aalborg, Denmark

**Keywords:** atrial fibrillation, obesity, paradox, body mass index, stroke, prognosis

## Abstract

**Background:**

The prognostic impact of obesity on patients with atrial fibrillation (AF) remains under-evaluated and controversial.

**Methods:**

Patients with AF from the Gulf Survey of Atrial Fibrillation Events (Gulf SAFE) registry were included, who were recruited from six countries in the Middle East Gulf region and followed for 12 months. A multivariable model was established to investigate the association of obesity with clinical outcomes, including stroke or systemic embolism (SE), bleeding, admission for heart failure (HF) or AF, all-cause mortality, and a composite outcome. Restricted cubic splines were depicted to illustrate the relationship between body mass index (BMI) and outcomes. Sensitivity analysis was also conducted.

**Results:**

A total of 1,804 patients with AF and recorded BMI entered the final analysis (mean age 56.2 ± 16.1 years, 47.0% female); 559 (31.0%) were obese (BMI over 30 kg/m^2^). In multivariable analysis, obesity was associated with reduced risks of stroke/systematic embolism [adjusted odds ratio (aOR) 0.40, 95% confidence interval (CI), 0.18–0.89], bleeding [aOR 0.44, 95%CI, 0.26–0.74], HF admission (aOR 0.61, 95%CI, 0.41–0.90) and the composite outcome (aOR 0.65, 95%CI, 0.50–0.84). As a continuous variable, higher BMI was associated with lower risks for stroke/SE, bleeding, HF admission, all-cause mortality, and the composite outcome as demonstrated by the accumulated incidence of events and restricted cubic splines. This “protective effect” of obesity was more prominent in some subgroups of patients.

**Conclusion:**

Among patients with AF, obesity and higher BMI were associated with a more favorable prognosis in the Gulf SAFE registry. The underlying mechanisms for this obesity “paradox” merit further exploration.

## Introduction

Atrial fibrillation (AF) is the most common sustained arrhythmia in clinical practice, with a prevalence of approximately 2–4% in adults (1). Attributing to the aging population (2), accumulation of risk factors (3), and the advancement of diagnostic techniques (4), the incidence of AF will continue to surge in the next decades (5, 6). AF significantly increases the risk of stroke (7), heart failure (HF)(8), dementia (9), and all-cause mortality (8), imposing a heavy burden on society and family (10) and healthcare costs (11).

As a well-known contributing factor to the development of AF (12–14), obesity has become a global problem in the past few decades. The number of overweight and obese people increased by 50% (from 26.5% in 1980 to 39% in 2015) and 80% (from 7% in 1980 to 12.5% in 2015), respectively, (15). However, the impact of obesity on AF prognosis is under-evaluated, and some studies suggest that weight loss can help reduce the burden of AF and delay AF progression (13, 16, 17). In contrast, other studies have indicated that the changes in body mass index (BMI) do not affect AF prognosis (18). Interestingly, obesity has also been associated with favorable AF prognosis in some reports (19–21), which is contrary to common sense and has been termed the “obesity paradox”(22).

Obesity is also prevalent in the Middle East region, which has attracted much attention recently (23). As revealed by a recent systematic analysis involving the Middle East region, the prevalences of obesity and overweight were 23% and over 30%, respectively,(24); which is comparative to the rest part of the globe, such as Asia (25), Europe (26), and America (27, 28).

However, insights into the prognostic role of obesity in patients with AF in this region are scarce. The present study aimed to investigate the prognostic impact of obesity and higher BMI on patients with AF in the Middle East Gulf region.

## Materials and methods

### The Gulf Survey of Atrial Fibrillation Events registry

In the present study, the Gulf Survey of Atrial Fibrillation Events (Gulf-SAFE) registry was applied. The Gulf-SAFE registry is a multicenter, prospective, and observational study with a 12-month follow-up, aimed at providing insights into AF management and outcomes in the Gulf region of the Middle East (29). The inclusion criteria and follow-up methods for the Gulf-SAFE registry have been previously published (29). Briefly, Gulf-SAFE included adult patients from 23 hospitals in 6 countries in the Middle East Gulf region who presented to the Emergency Room from 15th October 2009 to 30th June 2010 with AF on electrocardiogram or heart rhythm strips recorded over 30 s of adult patients. All patients who agreed to be included were required to sign an informed consent form. Exclusion criteria were patients who were not expected to be followed up regularly. This study excluded patients who died in hospital (*n* = 81), lacked height (*n* = 106) or weight data (*n* = 3) to calculate BMI, and those who did not complete 12 follow-up visits (*n* = 52), enabling analysis in a total of 1,804 patients. The treatment plan of the patients included in the study was decided by the treating physician, without the intervention of the investigator. Outpatient or telephone follow-up was performed at 1, 6, and 12 months after enrollment. The study protocols were approved by each national or institutional ethics committee.

### Statistical analysis

Continuous variables were expressed as mean and standard deviation, and categorical variables as frequencies and percentages. When comparing the differences between the two groups, the chi-square test was used for categorical variables, the independent samples *t*-test for normally distributed continuous variables and the Mann-Whitney U test for non-normally distributed continuous variables. The BMI was calculated as the body weight (Kg) divided by the square of height in meters. Obesity was defined as BMI ≥ 30 kg/m^2^. Per the protocol design of the Gulf SAFE registry, we do not have time-to-event variable, therefore we could only conduct risk-related analysis based on Logistic regression model. A logistic regression model was established to analyze the influence of obesity on AF prognosis, and the results were expressed as crude and adjusted odds ratio (aOR). The model was adjusted for the following covariates: sex, type of AF, hypertension, diabetes, coronary artery disease, HF, history of previous stroke or major bleeding, peripheral vascular disease, abnormal renal function, alcohol and smoking history, and baseline medications [aspirin, clopidogrel, angiotensin-converting enzyme inhibitors, angiotensin receptor blockers (ARBs), digoxin, statins, and warfarin]. The annual incidences of all available outcomes were calculated and expressed as per 100 patient years. Different BMIs were divided into four groups (BMI at < 25, 25–30, 30–35, and > 35 kg/m^2^) as ordered categorical variables, the incidence of different outcome events was the dependent variable, and the *P*-value obtained by Linear-by-Linear Association was the *P* for trend value. Restricted cubic splines were drawn for demonstrating the OR and 95% confidence interval (CI) of BMI for clinical outcomes, including stroke/systematic embolism (SE), bleeding (major or clinically relevant non-major bleeding), admission for AF or HF, all-cause mortality, and composite outcome events (a summary all above events). If any of the above-mentioned outcomes occurred, the composite outcome events were treated as positive. Sensitivity analysis was conducted to explore the impact of obesity on subgroups of patients with classified by gender, age, the presence of diabetes and hyperlipidemia. All analyses were performed using R (4.1.2 version). Two-sided *P* < 0.05 was considered statistically significant.

## Results

### Baseline characteristics

A total of 1,804 patients with AF (47.0% female, mean age 56.2 ± 16.1 years) were included in this study. According to the criteria of BMI ≥ 30 kg/m^2^, 559 people (31.0%) were identified as obese, of which 58.7% were female, and the average age was 59.1 ± 13.3 years. The clinical data and demographic characteristics of the patients are shown in Table 1.

**TABLE 1:** Baseline characteristics of obese and non-obese patients with atrial fibrillation.

Characteristics	Non-obese (*n* = 1,245)	Obese (*n* = 559)	*P*-value
Age (years)	55.0 ± 17.1	59.1 ± 13.3	**<0.001**
>65 yo	391 (31.4%)	204 (36.5%)	**0.034**
>75 yo	169 (13.6%)	68 (12.2%)	0.413
Female	519 (41.7%)	328 (58.7%)	**<0.001**
Height (cm)	165.3 ± 9.3	162.5 ± 9.4	**<0.001**
Weight (kg)	68.9 ± 11.8	94.2 ± 16.3	**<0.001**
Smoking	341 (27.4%)	92 (16.5%)	**<0.001**
Heavy drinking	3 (0.2%)	3 (0.5%)	0.38
SBP (mmHg)	129 ± 26	137 ± 25	**<0.001**
DBP (mmHg)	79 ± 16	83 ± 14	**<0.001**
Heart rate (bpm)	119 ± 32	123 ± 33	**<0.001**
AF-related ER visit	584 (46.9%)	295 (52.8%)	**0.02**
**Comorbidities**			
Hypertension	566 (45.6%)	387 (69.5%)	**<0.001**
Dyslipidemia	372 (30.0%)	264 (48.2%)	**<0.001**
Heart failure	361 (29.1%)	117 (20.9%)	**<0.001**
Coronary artery disease	343 (27.8%)	159 (28.7%)	0.701
Diabetes mellitus	300 (24.1%)	238 (42.7%)	**<0.001**
Rheumatic heart disease	231 (18.6%)	31 (5.6%)	**<0.001**
Stroke	98 (7.9%)	43 (7.7%)	0.896
Renal dysfunction	63 (5.1%)	28 (5.0%)	0.963
COPD	55 (4.4%)	34 (6.1%)	0.132
Thyroid disease	41 (3.3%)	52 (9.5%)	**<0.001**
Major bleeding	39 (3.1%)	15 (2.7%)	0.605
Sleep apnea	8 (0.6%)	13 (2.3%)	**0.002**
CHADS_2_ score	1.33 ± 1.31	1.66 ± 1.30	**<0.001**
CHA_2_DS_2_-VASc score	2.10 ± 1.73	2.64 ± 1.75	**<0.001**
HAS-BLED score	1.04 ± 1.03	1.19 ± 1.06	**0.006**
**Baseline medications**			
Beta-blocker	740 (59.4%)	322 (56.5%)	0.464
Aspirin	684 (54.9%)	316 (56.5%)	0.530
Warfarin	663 (53.3%)	306 (54.7%)	0.558
Diuretic	622 (50.0%)	245 (43.8%)	**0.016**
Statin	554 (44.5%)	323 (57.8%)	**<0.001**
Digoxin	491 (39.4%)	156 (27.9%)	**<0.001**
ACEI	478 (38.4%)	195 (34.9%)	0.154
ARB	140 (11.2%)	121 (21.6%)	**<0.001**
Clopidogrel	121 (9.7%)	72 (12.9%)	**0.045**
**Examinations**			
Left atrial diameter (mm)	44.2 ± 9.5	43.7 ± 7.4	0.366
LVEF (%)	50.3 ± 13.3	53.8 ± 12.1	**<0.001**
Left ventricular hypertrophy	308 (24.7%)	145 (25.9%)	0.587
Creatinine level (μmol/L)	108.7 ± 87.8	98.4 ± 79.9	**0.018**

*Obese was defined as body mass index over 30 kg/m^2^. ACEI, Angiotensin-converting enzyme Inhibitor; ARB, Angiotensin receptor blockers; COPD, chronic obstructive pulmonary disease; DBP, diastolic blood pressure; ER, emergency room; LVEF, Left ventricular ejection fraction; SBP, systolic blood pressure. This section may be divided by subheadings. It should provide a concise and precise description of the experimental results, their interpretation, as well as the experimental conclusions that can be drawn. Available data for respective characteristics were as follows: smoking (*n* = 1,793), hypertension (*n* = 1,799), dyslipidemia (*n* = 1,785), heart failure (*n* = 1,799), coronary artery disease (*n* = 1,787), diabetes mellitus (*n* = 1,799), rheumatic heart disease (*n* = 1,797), thyroid disease (*n* = 1,772). For other individual characteristic variables listed above, the available data was 1804. Bold letters represent *P* < 0.05 and are considered statistically significant.

Obese patients were older, more female, less likely to smoke, and had faster heart rates and higher blood pressure on presentation (all *P* < 0.05). In addition, patients with obesity had higher prevalences of hypertension, dyslipidemia, diabetes, thyroid disease, and sleep apnea (*P* < 0.05, respectively). Some comorbidities were less common in obese patients, such as HF and rheumatic heart disease (see Table 1). Obese patients had higher mean CHADS2 (1.66 ± 1.30 vs. 1.33 ± 1.31), CHA2DS2-VASc (2.64 ± 1.75 vs. 2.10 ± 1.73) and HAS-BLED (1.19 ± 1.06 vs. 1.04 ± 1.03) scores (all *P* < 0.05). Patients with obesity were more likely to receive statins, ARBs, and clopidogrel, but less likely to use diuretics and digoxin (see Table 1).

### Incidence of outcome events

**FIGURE 1:**
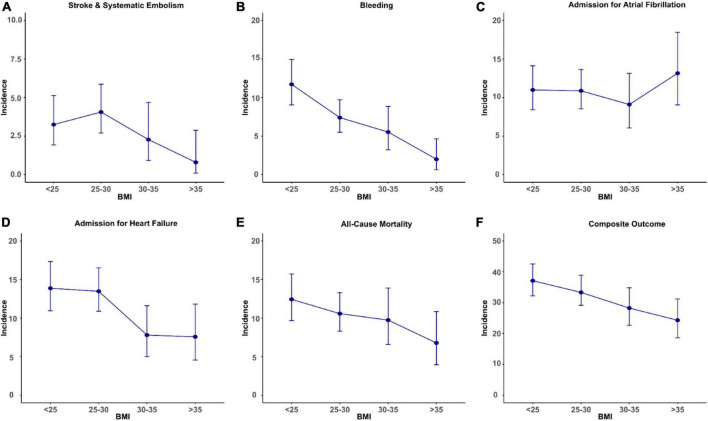
Body mass index and incident rates of clinical outcomes [**(A)** stroke and systematic embolism; **(B)** bleeding; **(C)** admission for atrial fibrillation; **(D)** admission for heart failure; **(E)** all-cause mortality; **(F)** composite outcome] per 100 patient-years. The bar represents confidence interval of incident rate.

During the 12-month follow-up period, there was a significant inverse association between BMI and the incidence of stroke/SE, bleeding, admission for HF, all-cause mortality, and the composite outcome showed a decreasing trend (see Table 2 and Figure 1) (*P* for trend < 0.001, respective), which were more prominent regarding the events of bleeding, HF admission, all-cause mortality, and the composite outcome. For patients with BMI at < 25, 25–30, 30–35 kg/m^2^, and > 35 kg/m^2^, the incidences of the composite outcome were 37.1 (32.2–42.6), 33.3 (29.2–38.9), 28.3 (22.6–34.8), and 24.3 (18.6–31.2) per 100 patient-years, respectively, (see Figure 1) (*P* for trend < 0.001).

**TABLE 2:** BMI category and outcome events.

	Stroke and SE (*n*/%)	Bleeding (*n*/%)	AF admission (*n*/%)	HF admission (*n*/%)	All-cause mortality (*n*/%)	Composite outcome (*n*/%)
<25 kg/m^2^ (*n* = 579)	19 (3.3)	65 (11.2)	62 (10.7)	79 (13.6)	69 (11.9)	208 (35.9)
25–30 kg/m^2^ (*n* = 667)	27 (3.0)	51 (7.6)	74 (11.1)	91 (13.6)	73 (10.9)	228 (34.2)
30–35 kg/m^2^ (*n* = 309)	7 (2.3)	17 (5.5)	28 (9.1)	25 (8.1)	30 (9.7)	88 (28.5)
>35 kg/m^2^ (*n* = 249)	2 (0.8)	6 (2.4)	33 (13.3)	18 (7.2)	17 (6.8)	60 (24.1)

### The outcome events in obese and non-obese patients

When dividing patients into obese and non-obese groups, obese patients were less likely to have stroke/SE [crude odds ratio (OR) = 0.43], bleeding (OR = 0.42), admission for HF (OR = 0.53), all-cause mortality (OR = 0.69), and the composite outcome (OR = 0.69) (*P* < 0.05, respectively). In the multivariable logistic regression model, after adjusting with other cofounders including medical history, baseline medication, etc., the result was generally the same for stroke/SE [adjusted OR (aOR) = 0.40], bleeding (aOR = 0.44), admission for HF (aOR = 0.61), and the composite outcome (aOR = 0.65) (all P < 0.05) (see Table 3).

**TABLE 3:** Odds ratio of clinical outcomes comparing obese and non-obese patients.

Clinical outcomes	Crude			Adjusted*		
	OR	95% CI	*P*-value	OR	95% CI	*P*-value
Stroke or SE	0.43	0.21–0.88	<0.001	0.40	0.18–0.89	0.024
Bleeding	0.42	0.26–0.66	<0.001	0.44	0.26–0.74	0.002
AF admission	0.99	0.73–1.38	0.994	0.93	0.67–1.30	0.678
HF admission	0.53	0.37–0.75	<0.001	0.61	0.41–0.90	0.013
All-cause death	0.69	0.49–0.97	0.035	0.69	0.47–1.03	0.066
Composite outcome	0.69	0.54–0.83	<0.001	0.65	0.50–0.84	0.001

AF, atrial fibrillation; CI, confidence interval; HF, heart failure; OR, odds ratio; SE, systemic embolism. *Adjusted for sex, type of AF, hypertension, diabetes, coronary artery disease, HF, history of previous stroke or major bleeding, peripheral vascular disease, abnormal renal function, alcohol and smoking history, and baseline medications (aspirin, clopidogrel, angiotensin-converting enzyme inhibitors, ARB, digoxin, statins, and warfarin).

### Odds ratio of body mass index as a continuous variable for outcomes

**FIGURE 2:**
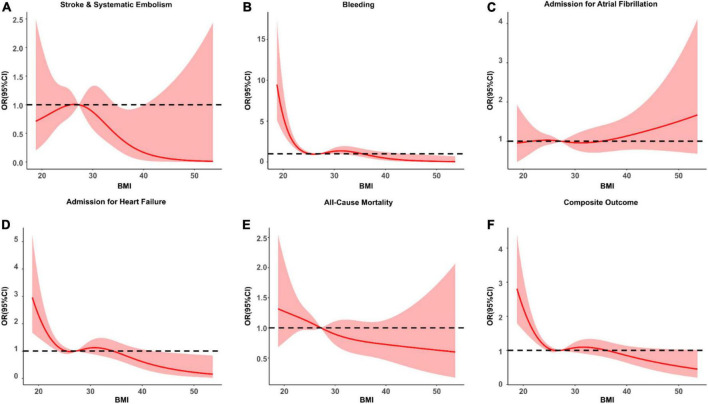
The hazard ratio (95% CI) BMI as a continuous variable for clinical outcomes [**(A)** stroke and systematic embolism; **(B)** bleeding; **(C)** admission for atrial fibrillation; **(D)** admission for heart failure; **(E)** all-cause mortality; **(F)** composite outcome]. The solid red line is the multivariate adjusted odds ratio, and the pink shade represents the 95% confidence interval. Unrelated reference lines are represented by dotted lines.

For illustrating the relationship between continuous BMI and AF prognosis, restricted cubic splines were depicted (see Figure 2). The risk of stroke/SE was highest at a BMI of 28 kg/m^2^, followed by a decreasing trend with a higher BMI. The risk of admission for AF did not show significant changes. While, the risks of bleeding, HF admission, all-cause mortality, and composite outcomes decreased with higher BMI (Figure 2).

### Sensitivity analysis for obesity in subgroups of patients

**FIGURE 3:**
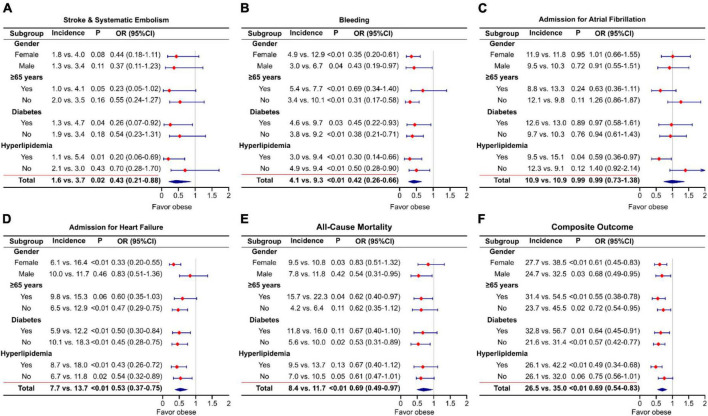
Odds ratio and forest plots of outcomes [**(A)** stroke and systematic embolism; **(B)** bleeding; **(C)** admission for atrial fibrillation; **(D)** admission for heart failure; **(E)** all-cause mortality; **(F)** composite outcome] in subgroups of patients.

To show the impact of obesity on outcomes in subgroups of patients, we have conducted a sensitivity analysis and depicted forest plots for comparison. For stroke/SE events, obesity showed a significantly lower OR in patients with hyperlipidemia (OR = 0.20) and diabetes (OR = 0.26). For bleeding events, obese patients had a significantly lower OR in all subgroups except in those aged ≥ 65 years. The risk of AF admission did not show significance. For HF admission, obesity showed lower OR for all subgroups except in males. For obesity, the risk of all-cause mortality was significantly lower in males (OR = 0.54), elderly (OR = 0.62), and non-diabetes (OR = 0.53) compared with the non-obese population. For the risk of composite outcomes, obesity showed significantly lower OR for all subgroups, except those without hyperlipidemia (see Figure 3).

## Discussion

This is the first study assessing the impact of obesity on the prognosis of AF patients from the Middle East Gulf region. We found that obesity is prevalent among AF patients, with nearly one-third of all enrolled subjects. Obesity was independently associated with lower risks of stroke/SE, bleeding, HF admission, all-cause mortality, and composite outcomes, consistent with what has been termed the “obesity paradox.” With the increase in BMI, the risks of these multiple outcome events showed a decreasing trend.

The prevalence of obesity is increasing in the general population and those with AF. In a recently published study in Spain which included 14,849 patients with AF, 46% of patients were obese (30). In the EORP-AF (EURObservational Research Programme AF) General Pilot Registry, among the 2,540 AF patients included, 42.7% were overweight and 29.0% were obese (18). This is roughly the same as the proportion in the present study. Obesity is one of the major contributors to the development of AF (3), and weight loss was associated with an improved prognosis for AF patients (17, 31). In the reports from Patti et al. (*n* = 9,330), obesity was associated with worse outcomes regarding thromboembolic and bleeding risks (32). Despite the well-established causal associations between obesity and AF, some studies have demonstrated controversial results suggesting a favorable effect of higher BMI. For instance, in the ARISTOTLE (Apixaban for Reduction in Stroke and Other Thromboembolic Events in Atrial Fibrillation) trial, which is a large prospective cohort study including 17,913 AF patients with at least one risk factor for stroke, BMI > 25 kg/m^2^ and high waist circumference (>102 cm for men, > 88 cm for women) were associated with lower risks of stroke and all-cause mortality (19). In a systematic analysis enrolling 13 randomized trials on AF, Proietti et al. also found evidence for an obesity paradox, whereby both overweight (OR 0.75, 95%CI, 0.66–0.84) and obese (OR 0.62, 95%CI, 0.54–0.70) patients had a lower risk for stroke/SE (21). The risk of major bleeding was lower in obese patients compared with normal-weight individuals (OR, 0.84; 95% CI, 0.72–0.98) (21).

In the present study, with increasing BMI, the risks of stroke/SE, bleeding risk, admission for HF, all-cause mortality, and the composite outcome were reduced; which is contrary to our common belief, and has been termed as “obesity paradox.” When treating BMI as a continuous variable as demonstrated by restricted cubic splines (Figure 2), we found that multiple outcome events showed higher risks when BMI is lower than < 28 kg/m^2^. More importantly, for the risk of stroke and SE, the highest risk was presented at the level of BMI = 28 kg/m^2^. Similar pattern was seen for the risk of AF admission. This phenomenon has attracted much attention in recent years (18, 19, 33, 34). In the ENGAGE AF-TIMI 48 study (*n* = 21,028), higher BMI (per 5 kg/m^2^ increase) was associated with lower risks of stroke/SE (hazard ratio = 0.88, *P* = 0.0001) and mortality (hazard ratio = 0.91, *P* = 0.0001) (33). In the EORP-AF study (*n* = 2,540), the risk of all-cause mortality in female overweight and obese AF patients was lower than that of normal-weight AF patients (18). Similarly, in a multicenter, longitudinal, observational study with 12 months of follow-up, which included 1,193 patients with AF, higher basal BMI was associated with a lower fatality rate (35). With a higher BMI category, mortality risk decreased by 26.4% (35). This was also seen in a generally well-anticoagulated AF population. In a recent large, multicenter, retrospective study involving 15 centers and 6,164 patients with AF on anticoagulants in China, higher BMI was negatively associated with major bleeding (OR = 0.353), total bleeding (OR = 0.664), and all-cause death (OR = 0.370) (36).

Interestingly, the obesity “paradox” is not only present in AF but also observed in coronary artery disease, HF, hypertension and other non-cardiovascular diseases (37–40). For instance, in the TOPCAT (Treatment of Preserved Cardiac Function Heart Failure with an Aldosterone Antagonist) trial with 1,749 patients with HF, both overweight (HR, 0.51; 95% CI, 0.27–0.95) and obesity (HR, 0.64; 95% CI, 0.43–0.98) were associated with reduced risk of all-cause death (41).

The phenomenon of high BMI increasing the risk of incident cardiovascular disease initially and reduces the risk of composite outcomes after cardiovascular disease occurred has been called the “obesity paradox”(22). However, it could be problematic to consider obesity as a protecting factor from the perspective of epidemiological standpoints. A reasonable explanation may be that obese patients tend to receive more aggressive treatment and follow-up regimens, which could improve cardiovascular disease prognosis (42). For instance, in the ARISTOTLE trial, the medication usage rates of statin and beta-blockers were 50%, and 68% for obese AF patients, compared to 34% and 56% for non-obesity patients (19). This phenomenon appears to be seen in the present study as well that obese patients received more statin and ARBs compared with non-obese individuals. Importantly, in the present study, obese patients were more treated with warfarin during follow-up time points. By categorizing patients into BMI < 30 kg/m^2^ and ≥ 30 kg/m^2^ groups, we found the following warfarin usage rates during follow-up: 48.9% vs. 51.7% (1st month), 42.8% vs. 47.4% (6th month), and 41.6% vs. 44.0% (12th month). This is a proof of the theory that obese patients received more treatment leading to better outcomes. In the present study, we found that obesity population were more prone to visit ER due to AF. Probably this is a reason why this group of patients were associated with more favorable AF-related outcome events. Further, in the Middle East region, relatively higher BMI may suggest better nutrition conditions and more available economical and medical resources, which could be transferred to better treatment selection. In addition, obese patients may receive more cardiorespiratory fitness exercise, which is beneficial in improving AF prognosis (43).

Nonetheless, BMI might not be an optimal index to describe obesity, which did not fully consider body composition, such as central obesity or visceral obesity. At present, some researchers have proposed the definition of metabolically healthy obese (MHO): systolic blood pressure < 130 mmHg and no antihypertensive drugs and waist-to-hip ratio < 0.95 (female)/1.03 (male) and self-reported without type 2 diabetes of obese people. The results showed that the cardiovascular risk of MHO patients was not significantly increased (44). This indicates that if we only use BMI to define obese, the prognosis judgment for some diseases could be misleading. The MHO may represent specialized adipose tissue composition with biologically protective functions (45).

### Limitations

Our study has several limitations. First, the body weight in this study was the data of the patients when they were included in the study, and the patients’ usual exercise and weight change trends were not recorded during the following follow-up period, which may have influenced our results. Second, despite the strict inclusion criteria and follow-up schedule of this study, its conclusions may be influenced by unmeasured and residual confounding factors, such as dietary habits and rest schedule. The Gulf SAFE registry did not provide the severity of AF during the enrollment procedure, which may have impact on AF prognosis. In addition, the anticoagulants used by the patients in this study did not include Non-vitamin K antagonists, and the quality of anticoagulation in the vitamin K antagonist population was not available, which would affect our assessment of AF prognosis, especially the stroke/SE and bleeding risk. Finally, one should not treat this as a distraction toward our endeavor of fighting obesity considering its culprit role in multiple cardiovascular disorders as recommended by guidelines (1). Ultimately, the contemporary management of AF needs a holistic or integrated care approach (46), which is recommended in guidelines (47), given the improved outcomes by adherence to such a strategy (48).

## Conclusion

In the Gulf-SAFE registry, obesity is prevalent among AF patients and associated with favorable outcomes. With the increase of BMI, the risks of stroke/SE, bleeding, admission for HF, all-cause mortality, and composite outcomes decreased significantly. The underlying mechanisms merit further investigation.

## Data availability statement

The data analyzed in this study was obtained from the authorization of PIs from individual center involved in this study. The datasets presented in this article are not readily available because of copyright. Requests to access the datasets should be directed to all PIs in this study.

## Ethics statement

The study protocols were approved by each National or Institutional Ethics Committee. The patients/participants provided their written informed consent to participate in this study.

## Author contributions

Y-GL and P-XX: drafting manuscript. GL and S-WL: designing study and editing manuscript. AA-A, WA, KS, NA, and MZ: participated in processing data and quality control. All authors have read and agreed to the published version of the manuscript.
